# Gut permeability may be associated with periprosthetic joint infection after total hip and knee arthroplasty

**DOI:** 10.1038/s41598-022-19034-6

**Published:** 2022-09-05

**Authors:** Emanuele Chisari, Jeongeun Cho, Marjan Wouthuyzen-Bakker, Javad Parvizi

**Affiliations:** 1 Rothman Orthopaedic Institute at Thomas Jefferson University, 125 S 9th St. Ste 1000, Philadelphia, PA 19107 USA; 2 Department of Medical Microbiology and Infection Prevention, University of Groningen, University Medical Center Groningen, Groningen, The Netherlands

**Keywords:** Gastrointestinal system, Medical research

## Abstract

A growing number of recent investigations on the human genome, gut microbiome, and proteomics suggests that the loss of mucosal barrier function, particularly in the gastrointestinal tract, may substantially affect antigen trafficking, ultimately influencing the close bidirectional interaction between the gut microbiome and the immune system. This cross-talk is highly influential in shaping the host immune system function and ultimately affecting the outcome of interventions. We hypothesized that the loss of mucosal barrier in the gut may be associatedto acute and chronic periprosthetic joint infections (PJI). Zonulin, soluble CD14 (sCD14), and lipopolysaccharide (LPS) were tested in plasma as part of a prospective cohort study of patients undergoing primary arthroplasty or revision arthroplasty because of an aseptic failure or PJI (as defined by the 2018 criteria). All blood samples were collected before antibiotic administration. Samples were tested using commercially available enzyme-linked immunosorbent assays as markers for gut permeability. A total of 134 patients were included in the study of which 44 patients had PJI (30 chronic and 14 acute), and the remaining 90 patients were categorized as non-infected that included 64 patients revised for aseptic failure, and 26 patients undergoing primary total joint arthroplasty. Both Zonulin (7.642 ± 6.077 ng/mL vs 4.560 ± 3.833 ng/mL; *p* < 0.001) and sCD14 levels (555.721 ± 216.659 ng/mL vs 396.872 ± 247.920 ng/mL; *p* = 0.003) were significantly elevated in the PJI group compared to non-infected cases. Higher levels of Zonulin were found in acute infections compared to chronic PJI (11.595 ± 6.722 ng/mL vs. 5.798 ± 4.841 ng/mL; *p* = 0.005). This prospective study reveals a possible link between gut permeability and the ‘gut-immune-joint axis’ in PJI. If this association continues to be borne out with a larger cohort and more in-depth analysis, it will have a clinically significant implication in managing patients with PJI. It may be that in addition to the administration of antimicrobials, patients with PJI and other orthopaedic infections may benefit from administration of gastrointestinal modulators such as pro and prebiotics.

## Introduction

Prior studies investigating the etiopathogenesis of surgical site infection (SSI) suggested three main avenue for infection to occur: local contamination occurring during surgery, hematogenous translocation of bacteria during concomitant bacteraemia, and contamination from adjacent infected tissues by the progression of the infective process^1^. While most of the research on SSI focused on minimizing any source of pathogens at the time of the surgery^2,3^, emerging evidence demonstrates how acute and chronic SSI can emerge more often from endogenous sources of microorganisms such as the gastrointestinal system, especially in the context of unbalanced gut flora, ‘dysbiosis’, and impaired gut permeability^4–8^.

One of the most common organisms causing periprosthetic joint infections (PJI), namely *Staphylococcus aureus* has been shown to be able to translocate from the gut to prosthetic joints or the surgical site in preclinical models of SSI and PJI^9,10^. An emerging theory, the 'Trojan Horse' hypothesis, assumes that the translocation process is not necessarily mediated by blood only (i.e. bacteremia), but also by blood cells such as neutrophils^11–13^ and macrophages^14^ that act as a" Trojan Horse" transporting pathogens between various sites. Mechanistic evidence supports a close interaction between pathogens like *S. aureus* and the immune system^15^, but confirmation of this phenomenon in vivo remains. Cohort studies, evaluating populations with high prevalence of gut microbiome dysbiosis, such as patients with obesity, reported higher risk of enterococcus and proteus infections, which are common gut commensals^16^.

Gut epithelium intercellular tight junctions (TJs) regulate paracellular antigen trafficking and microbiome-immune system interaction. TJs are highly dynamic structures that serve critical functions to maintain integrity of the intestinal epithelium under both physiological and pathological circumstances ^17–19^. Despite significant progress in our knowledge on the composition and function of the intercellular TJ, the mechanisms by which they are regulated remains largely unknown. One of the breakthroughs in understanding the role of gut permeability in health and disease has been the discovery of zonulin, the only physiologic intestinal permeability modulator described so far^20,21^. Zonulin has been used as a marker for increased intestinal permeability and is associated with soluble CD14 (sCD14) and lipopolysaccharide (LPS), other common markers of inflammation and bacterial translocations^22^. Thus, in combinations these markers allow for study of gut permeabilitym and can act as a proxy for gut microbiome and dysbiosis.

The hypothesis of the current study was that levels of Zonulin, LPS and sCD14, known indicators of gut permeability, will be elevated in patients with acute and chronic PJI indicating that some degree of gut impaired barrier function may be at associated to PJI.

## Materials and methods

After approval by the institutional review board of Thomas Jefferson University, and registration of the prospective study in clinicaltrial.gov (NCT04666519), patients scheduled for joint arthroplasty were screened for eligibility. Informed consent was obtained from all patients, and the study was conducted in accordance with relevant guidelines and regulations. Patients with the diagnosis of osteoarthritis undergoing primary total joint arthroplasty, and patients undergoing revision arthroplasty for aseptic failures or periprosthetic joint infection (PJI), as determined by the 2018 international consensus meeting (ICM) definition, were included^23^. Management of the patients was done according to standard of care. For revision patients, culture are collected with aseptic technique from multiple areas of the surgical site and then cultured according to good laboratory practices. Patients with prior history of joint infection, diagnosis of autoimmune diseases, inflammatory bowel disease, irritable bowel syndrome and otherwise unspecified chronic gut issues were excluded. Patients being included in the study were consented prior to obtaining blood samples. A total of 155 patients were screened and approached for inclusion, among whom 134 patients agreed to participate between January and November 2021. The blood samples were obtained by venepuncture and transported in citrate buffer vacutainers to the laboratory within a 40-min window. Once transported, samples were processed, split in cryovials and snap-frozen at − 80C freezer. Plasma samples were tested using validated ELISA assays for sCD14(Invitrogen sCD14 Human ELISA Kit), LPS (ENDONEXT™ EndoZyme® II—Recombinant Factor C (rFC) Endotoxin Detection Assay), and Zonulin (Human zonulin ELISA kit, Cusabio). The assay was performed in agreement with manufacturer instruction.

### Statistical analysis

Based on a medium effect size as a minimally clinical significant result (Cohen’s d = 0.5), a beta of 0.2, an alpha of 0.05, we determined that 128 patients were needed.

The data was tested for normality with the Shapiro–Wilk test. Data was then analyzed with parametric and non-parametric tests to answer study questions. Student t-test, Mann–Whitney test, ANOVA, and Kruskal–Wallis were used for continuous variables. A Chi-squared and Fischer exact test were used for categorical variables. To allow for easy readability, the figures were magnified. Pearson correlation test was used to draw correlation amont the continues variables normally distributed. Continuous variables were reported as mean and standard deviation or median and interquartile range depending on normal or not normal distribution.Full figure and data is available as Supplementary Material. Statistical analysis was performed using open-source software JASP, JASP Team (2020). JASP (Version 0.14.1)[Computer software].

## Results

Among the cohort of 134 (46% female) patients, with a mean age of 67.7 years (range, 36 to 92) and a mean BMI 30.6 ± 7.0 (Table 1), 44 patients underwent revision for PJI (30 chronic and 14 acute infections), 90 patients were classified as aseptic (26 primaries and 64 aseptic revisions).

**Table 1:** Baseline characteristics of the cohort.

Variable	Full cohort	ICM Negative	ICM positive
	*N* = 134	*N* = 90	*N* = 44
**Gender**			
Female	62 (46.3%)	41 (45.6%)	21 (47.7%)
Male	72 (53.7%)	49 (54.4%)	23(52.3%)
Age	67.7 (10.3)	67.7 (10.3)	67.614 (10.5)
Body mass index (Kg/m^2^)	30.6 (7.0)	30.6 (6.98)	30.8 (7.11)
**Joint**			
Hip	66 (49.3%)	47 (52.2%)	`19 (43.2%)
Knee	68 (50.7%)	43 (47.8%)	25 (56.8%)

ICM: International Consensus meeting definition for periprosthetic joint infection. For gender and joint variables, absolute number of subjects and rate as % was reported. For all other variables mean and standard deviation were reported.

**Figure 1:**
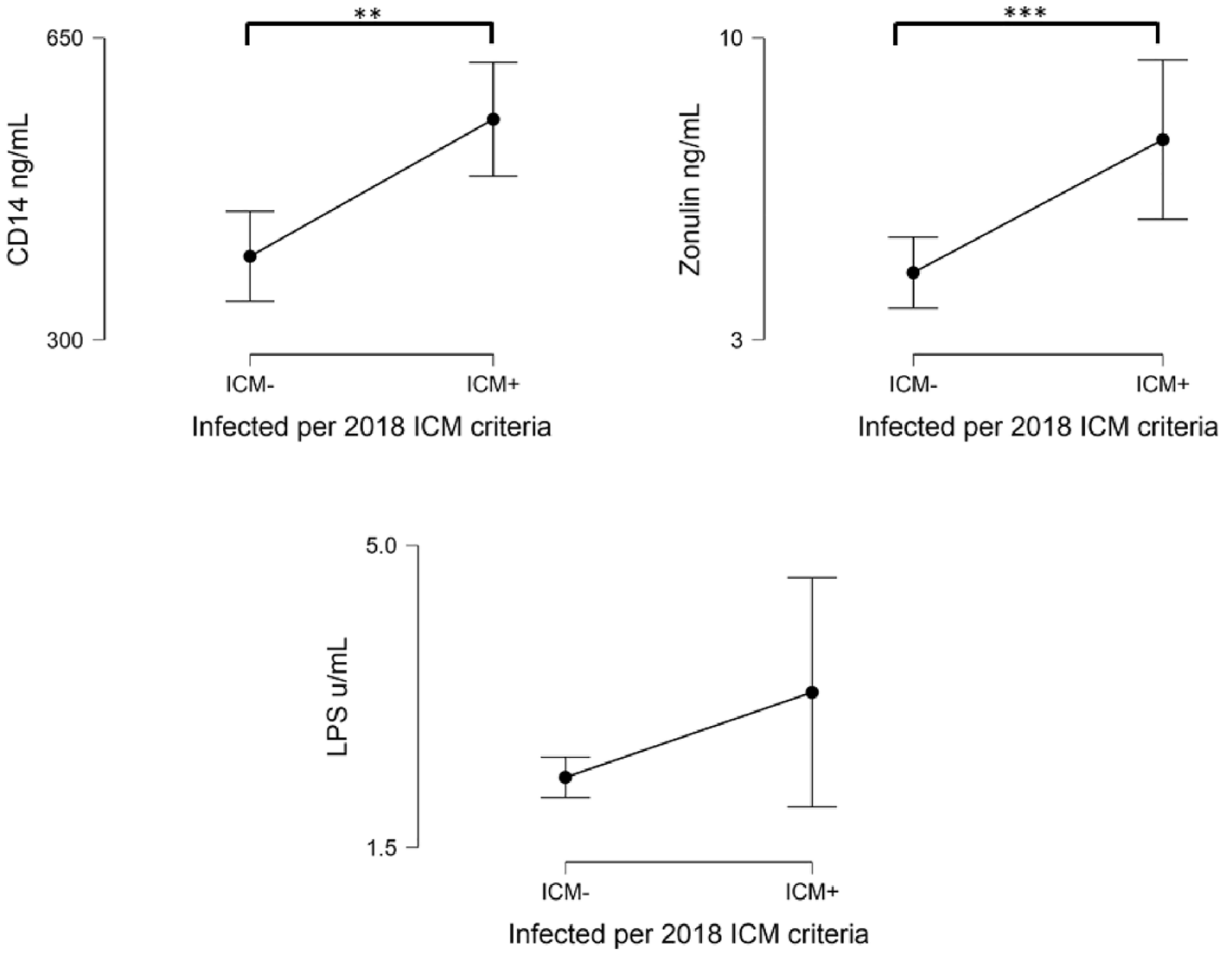
Descriptive plots of gut permeability biomarkers (CD14, Zonulin, LPS) are provided.Data were reported as mean and standard deviation. LPS: Lipopolysaccharide. ICM: International Consensus Meeting. **:*p* < 0.05; ***:*p* < 0.001. If data was not flagged (*), the comparison was not found significant.

No difference in the levels of LPS, sCD14 or Zonulin were retrieved between GRAM+ and GRAM− bacteria. Both Zonulin and sCD14, but not LPS, were found to be significantly elevated in the PJI group 7.642 ± 6.077 ng/mL and 555 ± 216 ng/mL, compared to non-infected cases (*p* < 0.001; *p* = 0.003) (Fig. 1).

For PJI cases, intraoperative-sampled tissue culture for bacterial identification was performed. Overall, out of the 44, 24 had culture positive, 17 were bacterial monomicrobial (14 GRAM +) 5 were polymicrobial, 1 was positive for fungi (*Candida Glabrata*) and one for not specified acid-fast positive bacilli (Supplementary material).

**Figure 2:**
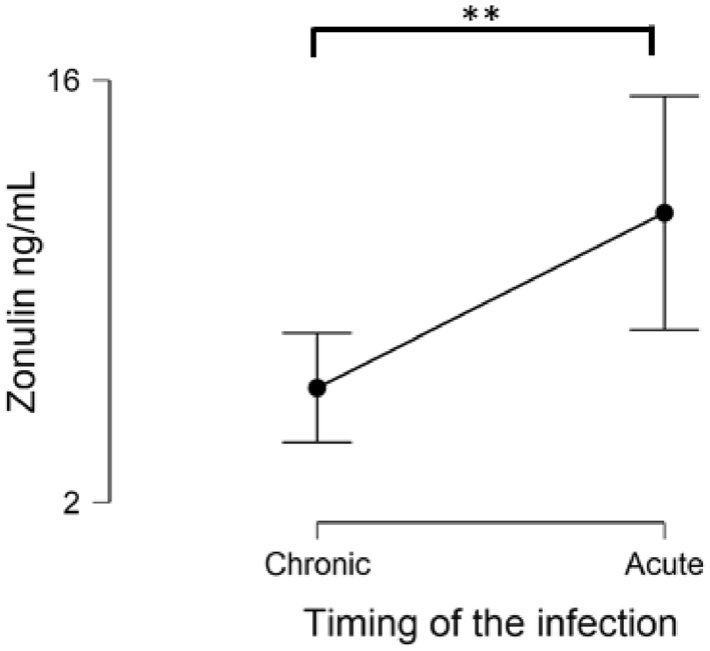
Levels of Zonulin based on the timing of the infection. **: *p* < 0.05.

When only patients categorized as PJI were evaluated, higher levels of Zonulin were found in acute infections compared to chronic (10.7 ± 6.2 ng/mL vs 5.8 ± 4.8 ng/mL; *p* = 0.005) (Fig. 2).

**Figure 3:**
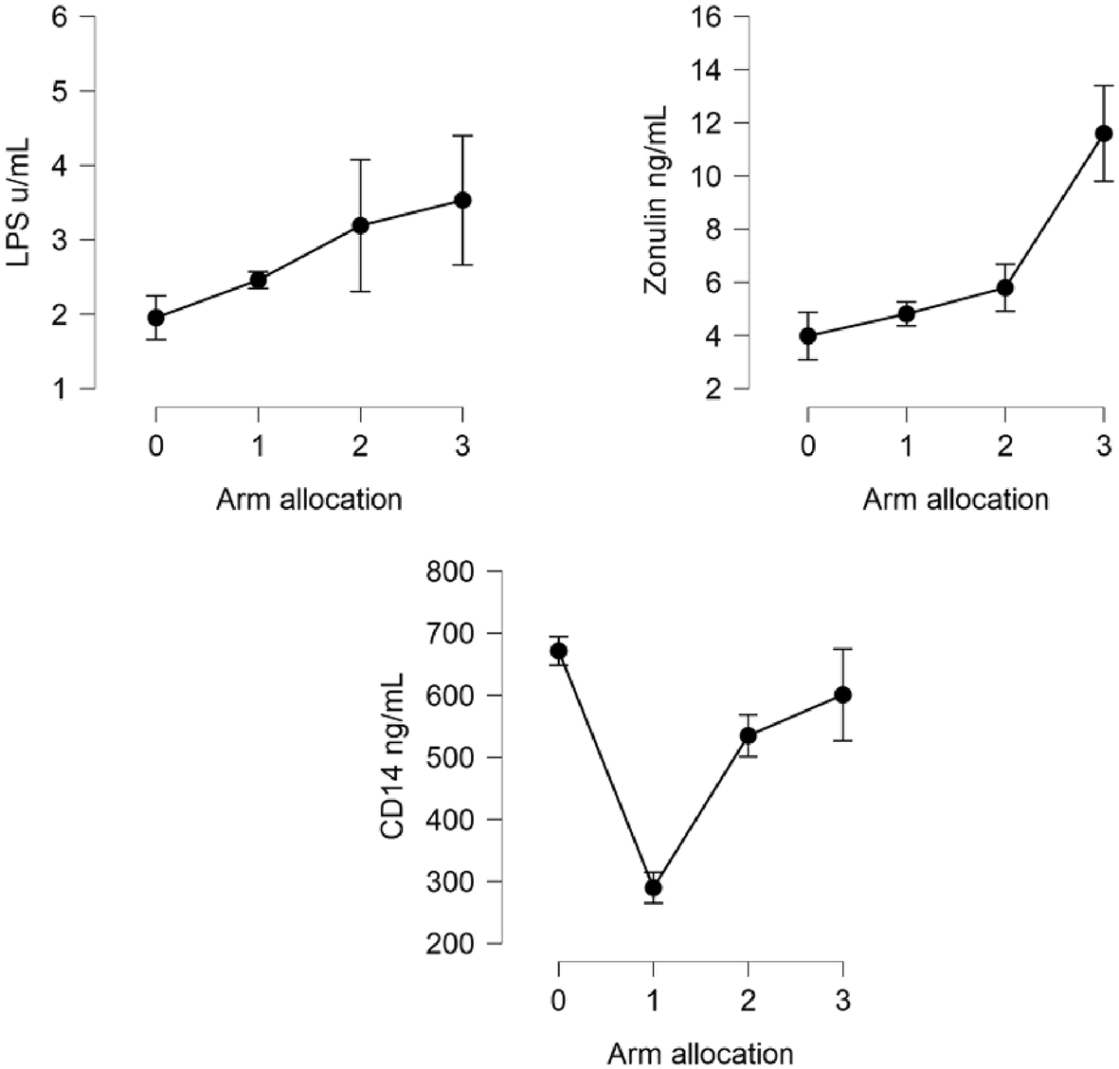
Descriptive plots based on arm allocation. Data are reported as mean and standard error. 0: primaries; 1: aseptic revisions; 2: Chronic PJI; 3:acute PJI.

When patients were allocated to the four different arms designed by this study enrollment plan, LPS and Zonulin showed increased levels for primaries, aseptic revisions, chronic and acute infections progressively (*p* < 0.001) (Table 2). Interestingly, sCD14 levels were significantly higher in primaries compared to aseptic revisions (Fig. 3).

**Table 2:** Plasma gut permeability markers based on arm allocation.

Variable	Primary arthroplasty	Aseptic revision	Chronic PJI	Acute PJI
	*N* = 26	*N* = 60	*N* = 30	*N* = 14
Zonulin ng/mL	3.980 (4.5)	4.811 (3.4)	5.798 (4.8)	11.595 (6.7)
CD14 ng/mL	671.424 (115.3)	289.625 (197.9)	534.781 (184.2)	600.591 (276.3)
LPS u/mL	1.95 (1.5)	2.45 (0.89)	3.19 (4.8)	3.53 (3.2)

Data was presented as mean and standard deviation.

PJI: periprosthetic joint infection. PJI definition was based on 2018 international consensus meeting definition.

**Figure 4:**
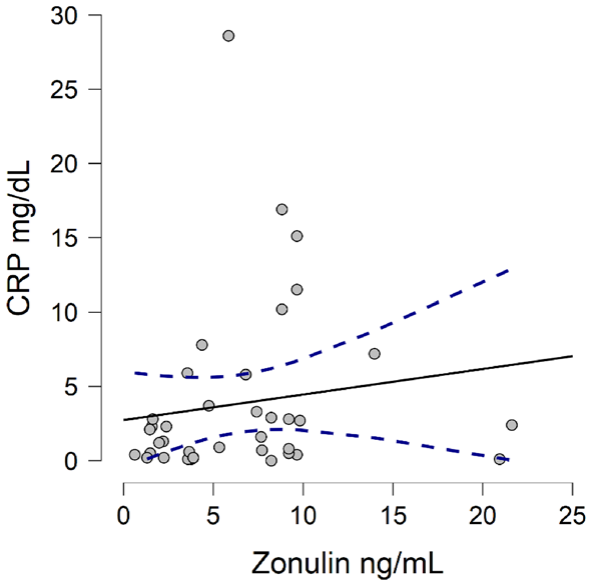
Correlation plot between Zonulin and C-reactive protein for infected patients.

No significant difference in the levels of Zonulin, sCD14 and LPS were found between males and females or hip and knees. Out of concern of correlation with acute-phase proteins, Zonulin and C-reactive protein (CRP) were investigated with no significant findings (Fig. 4).

## Discussion

Gut health and resident microbiome have been the focus of major research efforts in recent years around the globe. The emerging evidence suggests that there is a close connection between microbiome and overall host immunity and diseases^24–27^. The gut microbiome in particular has been found to modulate numerous diseases^28–30^. Studies have shown that patients with inflammatory bowel disease (IBD) for example, have a disturbed gut microbiome that could explicate the higher rate of systemic infections in these patients^31^. In a retrospective study from our institution, we also demonstrated that patients with IBD were at higher risk of developing periprosthetic joint infection (PJI) of hip and knee^30^. The prior findings was the impetus for the current study seeking further evidence for the presence of potential relationship between gut dysbiosis and PJI.

The current clinical study, first of its kind to our knowledge, adds weigh to prior pre-clinical studies demonstrating a relationship between gut dysbiosis and PJI in animal models^9,15,32^. We realize that surgical site infections such as PJI are complex diseases in which a vast number of etiological factors are in play^17,33–35^. We also recognize that a combination of environmental and genetic factors contribute to the development of any diseases such as PJI^1^. It is also clear that immune response plays a critical role in pathophysiology of any diseases, particularly infections^36,37^. Relying on the available evidence, we hypothesized that the gut microbiome and permeability have a role in shaping immune response to external pathogens as previously shown in other fields^17,37,38^. The preliminary, yet concrete, evidence generated by the current study brings to light the role that gut microbiome may play a role in PJI. As a matter of fact, the lack of association between microbiological profile of the infection and the levels of the gut permeability biomarker, make evident that their role is mostly associate with what happens in the gut microbiome and not in the local site of infection. We speculate that this association is due to the role of the human gut microbiome in shaping both adaptive and innate immune responses to external pathogens, that has been seen in other fields^24,25,27^.

An interesting, and somewhat surprising, findings of this study was that the level of sCD14 was lower in patients undergoing revision for aseptic failure than patients receiving primary joint replacement. While we cannot fully explain this finding, we speculate that the different behaviour of this biomarker is related to its physiological function. sCD14 is a marker of monocyte activation that acts as acute phase protein and its levels under the influence of LPS and other bacterial by-products^39,40^. Patients undergoing aseptic revision have been subject of two microbiome depleting stressors: antibiotic therapy and at least one previous surgery. Thus, it is possible that patients with gut imbalance (high level of Zonulin), but low biomass, can have a similar level of sCD14. To prove or refute such speculation metagenomic analysis of the gut microbiome in a longitudinal study will be needed.

Although the current study was prospectively designed, its exploratory nature is not exempt from limitations. First, this study is based on a single-center patient population and its generalizability may be limited. Although our cohort size was deemed to be adequate, based on power analysis, some differences in the level of biomarkers could be subject to type 2 statistical error, especially for small effect size interactions. Additionally, all the markers examined are proteins that behave as acute phase reactants, and their level have been shown to correlate with other biomarkers of inflammation^41,42^. As such, the absence of metagenomic data on this cohort does not allow definite conclusion on their pathophysiology. However, for the infected cohort, we did not find any correlation between Zonulin and CRP, a known biomarker of inflammation (Fig. 4). Finally, the biomarkers examined are indirect measure of gut epithelial barrier integrity, and direct measurement of gut dysbiosis and inflammation would require invasive testing which we did not perform. However, this approach seems scientifically reasonable as many prior studies have relied on indirect measure of gut dysbiosis to implicate its relationship with various diseases^17,19,43–48^.

In conclusion, the findings of this prospective ongoing study are interesting as a possible link between gut permeability, the ‘gut-immune-joint axis’, and PJI is being proposed. These findings are clinically relevant beseeching our attention to the role that gut modulators such as probiotics and prebiotics may play in the management of patients with PJI, who are routinely subjected to prolonged courses of antimicrobials. Future studies exploring the ‘gut-immune-joint axis’ and the role that modulators of gastrointestinal may play are needed.

## Supplementary Information

Supplementary Information.
