# Supplementary material for: Gut permeability may be associated with periprosthetic joint infection after total hip and knee arthroplasty
Source: Sci Rep. 2022 Sep 5;12:15094. doi: 10.1038/s41598-022-19034-6 (PMC9445168; doi:10.1038/s41598-022-19034-6)
Supplement: Supplementary file 1 — Supplementary Information. [file 41598_2022_19034_MOESM1_ESM.docx]

**Supplementary Materials**

**Figure S1.** Boxplot of plasma Zonulin of the full cohort


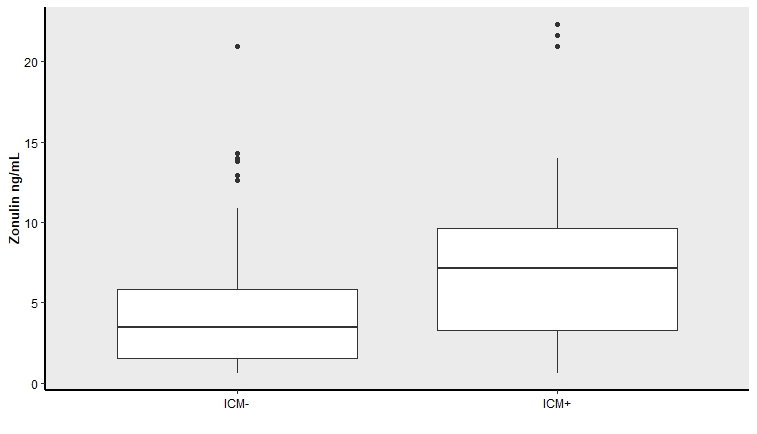


**Figure S2.** Scatterplot of plasma Zonulin of the full cohort


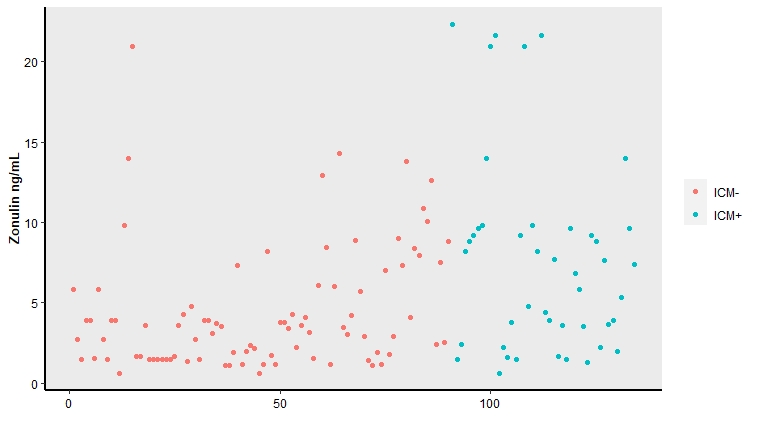


**Figure S3.** Boxplot of plasma sCD14 of the full cohort


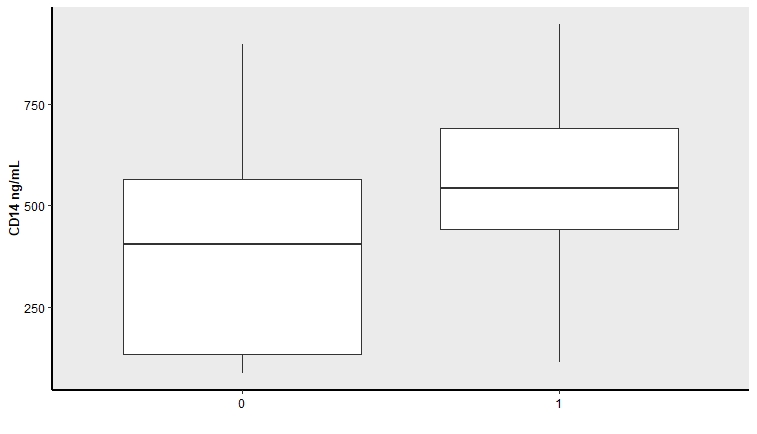


**Figure S4.** Scatterplot of plasma sCD14of the full cohort


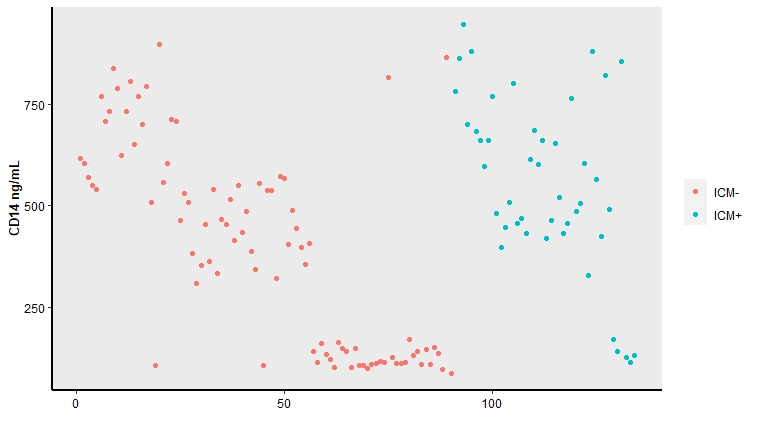


**Figure S5.** Boxplot of plasma Lipopolysaccharide of the full cohort


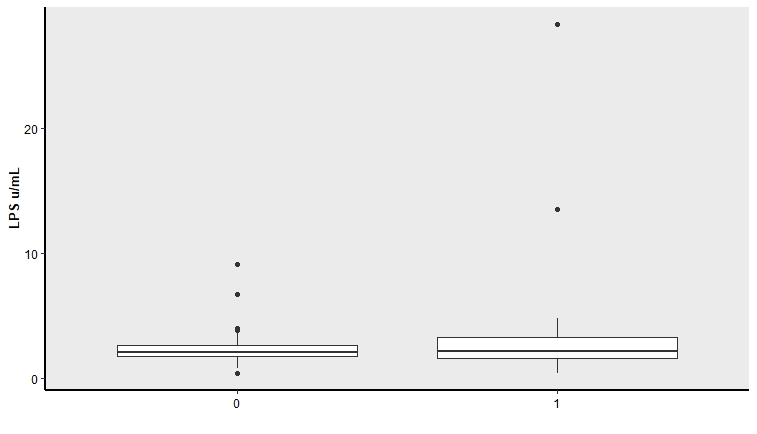


**Figure S6.** Scatterplot of plasma Lipopolysaccharide of the full cohort


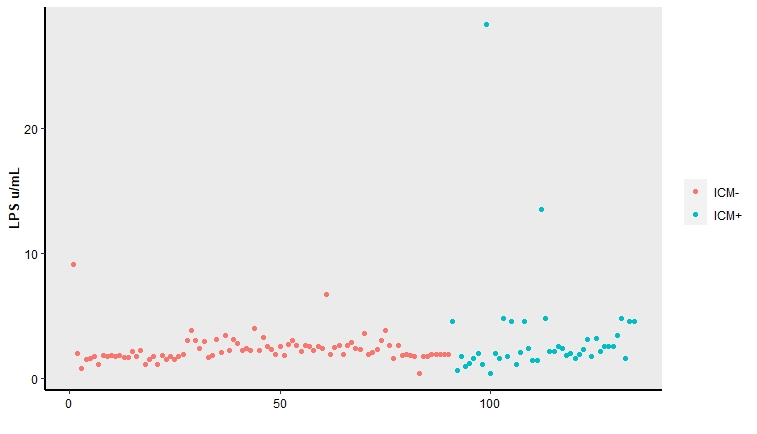


**Figure S7.** Boxplot of plasma Zonulin of the infected cohort based on timing**
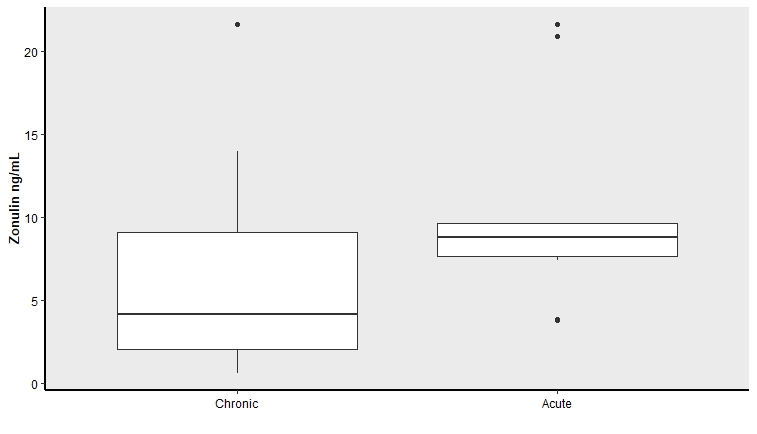
**
